# Factors Associated with the Use of Drug-Eluting Stents in Patients Presenting with Acute ST-Segment Elevation Myocardial Infarction

**DOI:** 10.1155/2015/528753

**Published:** 2015-05-28

**Authors:** Jose F. Chavez, Jacob A. Doll, Anuj Mediratta, Francesco Maffessanti, Janet Friant, Jonathan D. Paul, John E. A. Blair, Sandeep Nathan, Neeraj Jolly, Atman P. Shah

**Affiliations:** ^1^Section of Cardiology, Department of Medicine, The University of Chicago Medical Center, 5841 S. Maryland Avenue, Chicago, IL 60637, USA; ^2^Section of Cardiology, Department of Medicine, Rush University Medical Center, 1653 W. Congress Parkway, Chicago, IL 60612, USA

## Abstract

*Background*. Drug-eluting stents (DES) have proven clinical superiority to bare-metal stents (BMS) for the treatment of patients with ST-segment elevation myocardial infarction (STEMI). Decision to implant BMS or DES is dependent on the patient's ability to take dual antiplatelet therapy. This study investigated factors associated with DES placement in STEMI patients.* Methods*. Retrospective analysis was performed on 193 patients who presented with STEMI and were treated with percutaneous coronary intervention at an urban, tertiary care hospital. Independent factors associated with choice of stent type were determined using stepwise multivariate logistic regression. Odds ratio (OR) was used to evaluate factors significantly associated with DES and BMS.* Results*. 128 received at least one DES, while 65 received BMS. BMS use was more likely in the setting of illicit drug or alcohol abuse ([OR] 0.15, 95% CI 0.05–0.48, *p* ≤ 0.01), cardiogenic shock (OR 0.26, 95% CI 0.10–0.73, *p* = 0.01), and larger stent diameter (OR 0.28, 95% CI 0.11–0.68, *p* ≤ 0.01).* Conclusions*. In this analysis, BMS implantation was associated with illicit drug or alcohol abuse and presence of cardiogenic shock. This study did not confirm previous observations that non-White race, insurance, or income predicts BMS use.

## 1. Introduction

Drug-eluting stents (DES) have proven clinical superiority to bare-metal stents (BMS) in treatment of patients presenting with ST-segment elevation myocardial infarction (STEMI), primarily by reducing the need for repeat revascularizations [1–5]. Despite the data from these trials, many patients with STEMI still receive BMS. Previous studies have identified disparities in stent-type utilization by race and payor status [6–13]. These studies and others describe disparities in access to medical care and differing clinical outcomes with respect to race and socioeconomic status (SES), including treatment of cardiovascular disease [10, 14–16]. The causes of these disparities are multifactorial and the subject of much debate and ongoing research.

DES require at least 12 months of dual antiplatelet therapy (DAPT) to prevent stent thrombosis [17]. Nonadherence to DAPT is a major predictor of cardiac events and death following percutaneous coronary intervention (PCI) [17–21]. During primary PCI for the treatment of STEMI, the interventional cardiologist (IC) must make the decision to place DES or BMS, given the limited clinical information to predict patient compliance with DAPT. This decision must be made in a rapid fashion in order to adhere to current guidelines for timely revascularization.

The current guidelines recommend avoiding DES in the presence of financial barriers to continuing prolonged DAPT, social barriers that may limit patient compliance, or medical issues that involve bleeding risks or need for invasive procedures [5, 16]. Identification of pertinent financial and social barriers is left to the clinician, often in a situation that may not provide adequate time for thorough investigation. Two studies have retrospectively identified factors associated with drug nonadherence following PCI, with a proposed decision tool available to predict antiplatelet nonadherence [22, 23]. Nonetheless, factors that influence the physician's decision to use DES are incompletely understood. The purpose of this study was to identify demographic or clinical factors associated with DES placement in patients presenting with STEMI, specifically relevant to an urban population.

## 2. Methods

### 2.1. Study Design and Population

Baseline demographic, clinical data, and procedural characteristics for consecutive patients undergoing primary PCI with stent placement for STEMI at the University of Chicago Medicine were prospectively entered and retrospectively analyzed for a period from 2004 to 2012. Importantly for the context of this study, our Medical Center is a nonprofit, urban, tertiary care institution serving a predominately poor, uninsured, and African American population, which directly reflected the profile of the population of our study. After receiving approval from the Institutional Review Board, data were obtained from the index PCI procedural note, admission history and physical documentation, the hospital demographic database, and prior clinical notes in the electronic medical record (EMR). All analyzed data were readily available to the clinician at the time of PCI. Four ICs performed the procedures.

Race identification was self-reported upon admission; categories included African American, Caucasian, Middle Eastern, Asian, and Hispanic. Insurance status was categorized as Private, Medicare, Medicaid, or no insurance. Income was estimated by matching patient zip code at time of admission to US Census Bureau data for median household income by zip code. Presence or absence of a primary care physician or cardiologist at the treating institution was defined by a visit within the prior 6 months as noted in the EMR. Comorbid disease, medications, and illicit drug or alcohol abuse were obtained from the procedural note, admission history, and physical documentation, or the most recent clinical encounter note available to the IC at time of PCI. Patients were assigned to the DES group for analysis if they received at least one DES.

The particular stent chosen was at the discretion of the IC. BMS included Vision, Mini-Vision (Abbot Vascular, Temecula, CA), and Driver (Medtronic, Minneapolis, MN). DES included Taxus, Promus (Boston Scientific, Natick, MA), Cypher (Cordis, Miami, FL), Endeavor (Medtronic, Minneapolis, MN), Resolute (Medtronic, Minneapolis, MN), and Xience (Abbot Vascular, Temecula, CA). All patients received an antiplatelet and antithrombin regimen at the IC's discretion. Prior to the procedure, patients were given aspirin 325 mg and clopidogrel 300 mg to 600 mg, followed by daily aspirin 81 to 325 mg and clopidogrel 75 mg. Planned duration of antiplatelet therapy was at the operator's discretion and not assessed. Anticoagulation and other procedural details were also at the discretion of the operator.

### 2.2. Statistical Analysis

Data were described in the form of median with range and count with percentages for continuous or discrete variables, respectively. Bivariate analyses were performed using Fisher's exact or Chi-square statistics for categorical variables and logistic regression for continuous variables. Independent factors associated with choice of stent type were determined using stepwise multivariate logistic regression. Odds ratio (OR) with 95% confidence intervals (CI) were used to evaluate factors significantly associated with DES or BMS. A *p* value of <0.05 was considered statistically significant. Statistical analysis was performed using SPSS version 20.0 (IBM, Armonk, NY).

## 3. Results

A total of 193 patients presented with STEMI from October 2004 to February 2012 and were treated with primary PCI. Of these, 128 received at least one DES, while 65 received only BMS. The patients were predominately male (58%) and African American (82%) (Table 1). The median income of patients was $31,471, and significant portions of the patients were either uninsured (17%) or had Medicaid (12%) as the primary insurance (Table 1).

**Figure 1:**
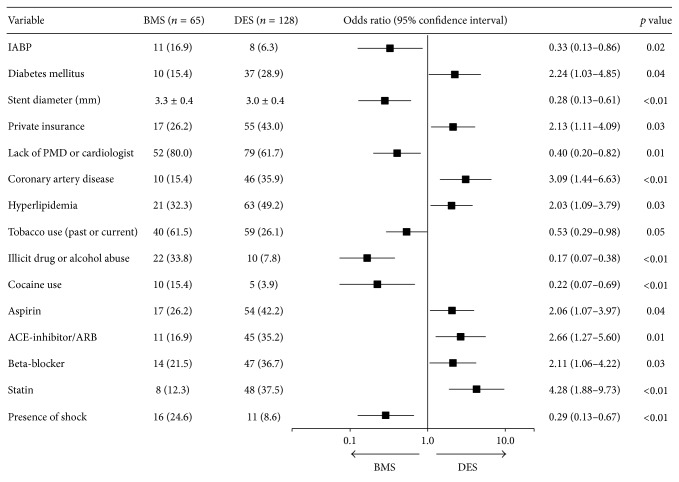
Bivariate logistic regression of factors associated with DES or BMS. Factors significantly associated with BMS use include the presence of shock, placement of an intra-aortic balloon pump (IABP), larger stent diameter, lack of primary care provider (PMD) or cardiologist, tobacco use, illicit drug or alcohol abuse, and cocaine use.

**Figure 2:**
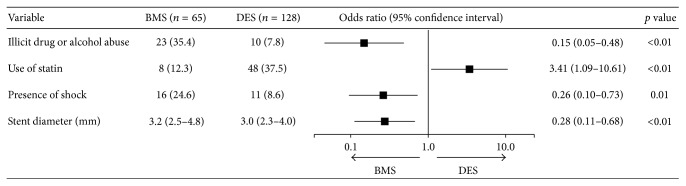
Multivariate logistic regression of factors associated with DES or BMS. Independent predictors of BMS use include the presence of shock, illicit drug or alcohol abuse, and larger culprit coronary artery stent.

There was no significant association with race, governmental insurance or lack of insurance, or income (Table 1). Association of procedural characteristics for use in primary PCI for acute STEMI is detailed in Table 2, with only stent diameter being significantly associated. Of note, all instances of abrupt closure were successfully intervened upon and thus considered a successful procedure. On bivariate analysis, factors significantly associated with DES use included: diabetes, private insurance, coronary artery disease (CAD), hyperlipidemia, aspirin use, ACE-inhibitor or ARB use, beta-blocker use, and statin use (Figure 1). Factors significantly associated with BMS use included the presence of shock, placement of an intra-aortic balloon pump (IABP), larger stent diameter, lack of PMD or cardiologist, tobacco use (past or present), illicit drug or alcohol abuse, and cocaine use (Figure 1). Multivariate logistic regression analysis identified presence of shock, all illicit drug (i.e., including cocaine use) or alcohol abuse and larger culprit coronary artery stent as independent predictors for BMS use (Figure 2). Use of statin was the only independent predictor for DES use (Figure 2).

## 4. Discussion

In this single center analysis with a primarily urban population, we found that the choice of BMS use in acute STEMI was associated with illicit drug or alcohol abuse and presence of cardiogenic shock. We also found that increased use of HMG-CoA reductase inhibitors (statins) was conversely associated with DES use. These differences remained significant after multivariable adjustment. This study did not confirm previous observations that non-White race, government or lack of insurance, or income predicts BMS use. Our study is unique in that it examines only PCI for STEMI, a relatively homogenous sample with regard to pathology and acuity. We believe this study more closely reflects physician decision-making as influenced by patient factors and examines clinical factors not previously included in larger registry analyses.

Many studies have demonstrated disparities in access to cardiovascular procedures and outcomes [6–8]. There is robust data that document that African American patients are less likely to receive invasive cardiac procedures and have worse cardiovascular outcomes [8, 9, 24]. In evaluating disparities, the decision to utilize DES versus BMS use during PCI focuses on the influence of patient factors on physician decision-making, while minimizing the impact of systemic barriers to care. In addition, revascularization must be achieved as rapidly as possible, limiting detailed investigation of a patient's likely adherence to DAPT. Previous studies, that include both multicenter and national registry analyses, have demonstrated that African American race, low SES, and those with poor insurance status are less likely to receive DES when PCI is indicated [10–13, 25–28]. Further, a majority of this data was collected during the time of the so-called DES era.

While previous studies suggest that race, SES, and insurance status predict compliance with DAPT, these factors have not been consistently associated with levels of adherence [29]. Our study population is unique in that it is primarily urban and poor, with a significant number lacking insurance. In this setting, our ICs have to use other factors to predict likelihood of adherence. Our analysis revealed that illicit drug or alcohol abuse, presence of cardiogenic shock, and larger culprit coronary artery (i.e., stent diameter) were independent factors associated with BMS. While a larger culprit coronary artery may also predicate BMS use, given in-stent restenosis would unlikely compromise flow as much as in a smaller caliber artery, the remaining factors reported in our study were significantly associated with BMS use. It has been shown that active substance abusers demonstrate particularly poor adherence to medical therapy, whereby the abuse compromises the effective treatment of other diseases [30]. Substance abusers may also suffer from psychiatric disorders, low SES, and homelessness, which is also likely to lead to poor compliance with prescribed therapies [29, 30]. Further, the presence of cardiogenic shock may result in increased BMS use because of the potential need for surgical revascularization, aggressive cardiopulmonary support, or a poor prognosis.

Increased use of statins was conversely associated with DES use in our analysis. Statin use may be a marker of medication adherence and compliance with treatment regimens that may have resulted in the increased use of DES. In the bivariate analysis, use of antiplatelet medication, ACE-inhibitor or ARB, and beta-blocker was also associated with DES use, further supporting the hypothesis that prior medication adherence may play a role in physicians choosing DES.

Our study was not designed to determine if this strategy of stent choice is appropriate or lead to improved outcomes. Guidelines which recommend avoiding DES in the setting of financial or social barriers to prolonged DAPT are based on numerous studies indicating increased incidence of stent thrombosis and death following early discontinuation of DAPT in patients with DES [18–21]. This is less prevalent among patients with implantation of BMS [20, 21]. Guidelines for prospectively identifying the nonadherent patient are lacking however. Prior studies have identified a variety of psychological, cognitive, and systemic barriers to medication adherence [29]. Recently, Quadros et al. identified factors associated with thienopyridine adherence among several hundred patients presenting for coronary stent implantation [23]. The authors proposed a risk score that included low income, unmarried status, no private insurance, acute coronary syndrome, and absence of diabetes as predictors of nonadherence. While a validated decision tool may one day be available, our study may help provide a more current snapshot of the IC decision-making at the time of PCI in STEMI.

### 4.1. Study Limitations

The limitations to our study include its relatively small sample size, which could have masked the potential effects of race on the choice of stent. However, power analysis showed that for proportions of stent type to race found in our study to become significant (*p* < 0.05), the total number of patients would need to be over 8000, which is unreasonable for a single center study of this nature. Other limitations are the single-site, retrospective design, and lack of outcome data. We are unable to exclude the influence of confounding variables that may have a greater impact on stent choice. The use of median income by zip code is a gross estimation of actual income and may result in misclassification. Further, there may be a misclassification of race as defined by current federal guidelines. The data is self-reported, categories are mutually exclusive, race and ethnicity are reported as separate categories, and more than one race may be selected. Also of importance, the baseline demographics for the previous studies highlighting race- or socioeconomic-based disparities influencing stent type included a mostly Caucasian, nonpoor, and insured population [10, 12, 25–28]. While our findings did not discover similar disparities, our population size (<200 patients) and predominately African American race may have affected our findings. Despite these limitations, we believe our study contributes to a more nuanced understanding of current DES use during the so-called DES era.

## 5. Conclusions

In an urban, tertiary care, university-affiliated institution, with a patient population of largely low SES, this study did not confirm previous observations that non-White race, government or lack of insurance, or income predicts BMS use. The choice of BMS implantation was instead associated with illicit drug or alcohol abuse and presence of cardiogenic shock, factors that may negatively influence a physician's confidence in a patient's adherence to DAPT. We believe these findings should promote further work to improve the prediction of medication adherence and appropriate stent choice in patients undergoing PCI.

## Figures and Tables

**Table 1:** Association of clinical variables with DES or BMS use in primary PCI for STEMI.

Variable (at time of PCI)	Bare-metal stent (*n* = 65)	Drug-eluting stent (*n* = 128)	*p* value
*Demographic *			
Age (years)	62 (34–99)	59 (27–88)	0.34
Gender (male)	41 (63.1)	70 (54.7)	0.28
Race (African American)	56 (86.2)	103 (80.5)	0.42
Income by ZIP code	28026 (15866–100377)	31571 (14205–100377)	0.11
Insurance status			0.11
No insurance	15 (23.1)	18 (14.1)	0.16
Medicaid	9 (13.8)	15 (11.7)	0.65
Medicare	24 (36.9)	40 (31.3)	0.52
Private	17 (26.2)	55 (43.0)	0.03
Lack of PMD or cardiologist	52 (80.0)	79 (61.7)	0.01
*Past medical history *			
Coronary artery disease	10 (15.4)	46 (35.9)	<0.01
Prior revascularization	11 (16.9)	38 (29.7)	0.15
Diabetes	10 (15.4)	37 (28.9)	0.05
Hypertension	46 (70.8)	93 (72.7)	0.87
Hyperlipidemia	21 (32.3)	63 (49.2)	0.03
Chronic renal insufficiency	8 (12.3)	8 (6.3)	0.17
Cancer (any)	6 (9.2)	6 (4.7)	0.22
Tobacco use (past or current)	40 (61.5)	59 (26.1)	0.05
Illicit drug (all) or alcohol abuse	23 (35.4)	10 (7.8)	<0.01
*Medications *			
Aspirin	17 (26.2)	54 (42.2)	0.04
Plavix	3 (4.6)	18 (4.1)	0.05
Warfarin	5 (7.7)	3 (2.3)	0.12
ACE-inhibitor/ARB	11 (16.9)	45 (35.2)	0.01
Beta-blocker	14 (21.5)	47 (36.7)	0.03
Statin	8 (12.3)	48 (37.5)	<0.01

Data expressed as median (range) or count (%) for continuous or discrete variables, respectively.

*p* value refers to DES versus BMS comparisons.

DES = drug-eluting stents.

BMS = bare-metal stents.

PCI = percutaneous coronary intervention.

STEMI = ST-segment elevation myocardial infarction.

ACE-inhibitor = angiotensin-converting-enzyme inhibitor.

ARB = angiotensin receptor blocker.

**Table 2:** Association of procedural characteristics for use in primary PCI for STEMI.

Variable (at time of PCI)	Bare-metal stent (*n* = 65)	Drug-eluting stent (*n* = 128)	*p* value
Stent placed ≥ 1	31 (47.7)	47 (36.7)	0.21
Multivessel PCI^†^	5 (7.7)	5 (4.0)	0.31
Primary lesion location			
Right coronary	29 (44.6)	41 (32.0)	0.11
Left circumflex	11 (16.9)	23 (18.0)	1.00
Left anterior descending	29 (44.6)	61 (44.7)	0.77
Presence of shock	16 (24.6)	11 (8.6)	0.01
Use of IVUS	4 (6.2)	8 (6.3)	1.00
Door-to-balloon < 90 min^*∗*^	37 (57.8)	65 (54.6)	0.76
AHA lesion A/B1^†^	13 (20.0)	37 (29.4)	0.22
AHA lesion B2/C^†^	52 (80.0)	89 (70.6)	0.22
Thrombectomy device^†^	24 (36.9)	36 (28.6)	0.25
Dissection^†^	4 (6.2)	4 (3.2)	0.45
Stent diameter (mm)	3.0 (2.5–4.8)	3.0 (2.3–4.0)	<0.01
Stent length (mm)	24.0 (9.0–108.0)	24.0 (8.0–81.0)	0.90
Successful procedure	65 (100.0)	128 (100.0)	1.00
Abrupt closure^†^	1 (1.5)	2 (1.6)	1.00
GP 2b/3a inhibitors^†^	44 (67.7)	77 (61.1)	0.43

^*∗*^Data missing in 10 cases (1 BMS, 9 DES).

^†^Data missing in 2 cases (2 DES).

Data expressed as median (range) or count (%) for continuous or discrete variables, respectively.

*p* value refers to BMS versus DES comparison, Mann-Whitney *U* test, or Fisher's exact test for continuous or discrete variables, respectively.

PCI = percutaneous coronary intervention.

STEMI = ST-segment elevation myocardial infarction.

IVUS = intravascular ultrasound.

## Abbreviations

HMG-CoA: 3-Hydroxy-3-methylglutaryl-coenzyme A
DES: Drug-eluting stent(s)
BMS: Bare-metal stent(s)
STEMI: ST-segment elevation myocardial infarction
PCI: Percutaneous coronary intervention
OR: Odds ratio
CI: Confidence interval
SES: Socioeconomic status
DAPT: Dual antiplatelet therapy
IC: Interventional cardiologist
EMR: Electronic medical record
CAD: Coronary artery disease
IABP: Intra-aortic balloon pump
PMD: Primary care provider
ACE-inhibitor: Angiotensin-converting-enzyme inhibitor
ARB: Angiotensin receptor blocker.
